# Hormonal Regulation of MicroRNA Expression in Steroid Producing Cells of the Ovary, Testis and Adrenal Gland

**DOI:** 10.1371/journal.pone.0078040

**Published:** 2013-10-28

**Authors:** Zhigang Hu, Wen-Jun Shen, Yuan Cortez, Xudong Tang, Li-Fen Liu, Fredric B. Kraemer, Salman Azhar

**Affiliations:** 1 Geriatric Research, Education and Clinical Center, VA Palo Alto Health Care System, Palo Alto, California, United States of America; 2 Division of Endocrinology, Stanford University, Stanford, California, United States of America; 3 Division of Gastroenterology and Hepatology, Stanford University, Stanford, California, United States of America; IPMC, CNRS UMR 7275 UNS, France

## Abstract

**Background:**

Given the emerging roles of miRNAs as potential posttranscriptional/posttranslational regulators of the steroidogenic process in adrenocortical and gonadal cells, we sought to determine miRNA profiles in rat adrenals from animals treated with vehicle, ACTH, 17α-E2 or dexamethasone. Key observations were also confirmed using hormone (Bt_2_cAMP)-treated mouse Leydig tumor cells, MLTC-1, and primary rat ovarian granulosa cells.

**Methodology:**

RNA was extracted from rat adrenal glands and miRNA profiles were established using microarray and confirmed with qRT-PCR. The expression of some of the hormone-sensitive miRNAs was quantified in MLTC-1 and granulosa cells after stimulation with Bt_2_cAMP. Targets of hormonally altered miRNAs were explored by qRT-PCR and Western blotting in adrenals and granulosa cells.

**Results:**

Adrenals from ACTH, 17α-E2 and dexamethasone treated rats exhibited miRNA profiles distinct from control animals. ACTH up-regulated the expression of miRNA-212, miRNA-182, miRNA-183, miRNA-132, and miRNA-96 and down-regulated the levels of miRNA-466b, miRNA-214, miRNA-503, and miRNA-27a. The levels of miR-212, miRNA-183, miRNA-182, miRNA-132, miRNA-370, miRNA-377, and miRNA-96 were up-regulated, whereas miR-125b, miRNA-200b, miR-122, miRNA-466b, miR-138, miRNA-214, miRNA-503 and miRNA27a were down-regulated in response to 17α-E2 treatment. Dexamethasone treatment decreased miRNA-200b, miR-122, miR-19a, miRNA-466b and miRNA27a levels, but increased miRNA-183 levels. Several adrenal miRNAs are subject to regulation by more than one hormone. Significant cAMP-induced changes in certain miRNAs were also noted in MLTC-1 and granulosa cells. Some of the hormone-induced miRNAs in steroidogenic cells were predicted to target proteins involved in lipid metabolism/steroidogenesis. We also obtained evidence that miR-132 and miRNA-214 inhibit the expression of SREBP-1c and LDLR, respectively.

**Conclusion:**

Our results demonstrate that expression of a number of miRNAs in steroidogenic cells of the testis, ovary and adrenal glands is subject to hormonal regulation and that miRNAs and their regulation by specific hormones are likely to play a key role in posttranscriptional/posttranslational regulation of steroidogenesis.

## Introduction

Steroid hormones, which are synthesized most prominently in the adrenal gland and gonads [1]–[3], play important roles in the regulation of carbohydrate, lipid and protein metabolism and immune function (glucocorticoids), salt and water balance and blood pressure regulation (mineralocorticoids) and maintenance of secondary sex characteristics, reproductive functions and muscle and bone growth (testosterone, progestins and estrogens) [4]. Steroidogenesis or biosynthesis of steroid hormones represents a complex multistep and multienzymes process by which precursor cholesterol is converted to pregnenolone and subsequently metabolized into other biologically active steroids in a tissue specific manner [1]–[4]. This process can be broadly divided into five major steps: 1) acquisition of cholesterol from exogenous (lipoproteins) and endogenous (*de novo* synthesis) sources for storage in the form of cholesterol esters (CEs) in lipid droplets, 2) mobilization of cholesterol from lipid droplet stored CEs, 3) transport of cholesterol to and from the outer mitochondrial membrane (OMM) to the inner mitochondrial membrane (IMM), where cytochrome P450 side chain cleavage enzyme (P450scc, encoded by CYP11A1) is localized, 4) P450scc catalyzed cleavage of a 6-carbon unit from the cholesterol side chain producing pregnenolone, the common precursor - for the synthesis of all of the other steroid hormones, and 5) efflux of pregnenolone from the mitochondria to the endoplasmic reticulum (ER), where it is converted by ER enzymes into intermediate precursors, which further shuttle between mitochondria and ER for the tissue specific production of progestins, estrogens, androgens, glucocorticoids or mineralocorticoids [2], [5].

Adrenal and gonadal steroidogenesis is predominantly controlled by trophic hormones (ACTH and LH/FSH, respectively) and is subject to both acute [3], [6]–[9] and chronic regulation [2], [3], [10]–[13]. Acute steroid synthesis that occurs over minutes in response to trophic hormone stimulation is controlled at the level of cholesterol delivery to the IMM for the first enzymatic step in the pathway, the conversion of cholesterol to pregnenolone by the P450scc. This rate limiting step, i.e., cholesterol transfer from OMM to IMM, is dependent upon the trophic hormone stimulated rapid induction (increased transcription) of the steroidogenic acute regulatory protein (StAR) [3], [14]–[16]. Although, the exact mechanism of action of StAR protein in mediating the cholesterol transfer across the mitochondrial membrane is not known, increasing evidence now suggests that StAR works in concert with several other proteins including peripheral benzodiazepine receptor (PBR)/18-kDa transporter protein (TSPO), voltage-dependent anion channel 1(VDAC1), phosphate carrier protein, cAMP-dependent protein kinase 1α (PKA-RIα) and TSPO-associated acyl-coenzyme A binding domain containing 3 (ABCD3) protein by forming a protein complex on the OMM [3], [16]. Chronic stimulation (hours to days) occurs through the induction of P450scc gene transcription leading to increased P450scc and consequent increased steroidogenic capacity [3], [10]–[13].

Thus, while significant information is currently available about the transcriptional regulation of both acute and chronic phases of steroidogenesis, relatively very little is known about the posttranscriptional and posttranslational regulation of steroidogenesis. The only exceptions are the phosphorylation-mediated modulation of StAR protein activity [17] and phosphorylation dependent activation of hormone-sensitive lipase (HSL) [18] during hormone-induced mobilization of stored cholesterol esters to supply precursor cholesterol for steroidogenesis. Few other downstream enzymes involved in steroidogenesis are also regulated by phosphorylation/dephosphorylation or by allosteric mechanisms [5]. Recently our laboratory has shown that scavenger receptor class B, type I (SR-BI), an HDL receptor that mediates bulk delivery of HDL-derived CEs into the steroidogenic cells of the adrenal gland and ovary (and to testicular Leydig cells under certain conditions) [2], is also subject to posttranscriptional/posttranslational regulation [19], [20].

MicroRNAs (miRNAs) comprise a novel class of endogenous non-protein-coding single-stranded small RNAs approximately 22–25 nucleotides long that have emerged as key posttranscriptional regulators of gene expression [21]–[25]. They are transcribed in the nucleus by RNA polymerase II into primary transcripts (pri-miRNAs) and then processed sequentially in the nucleus and cytoplasm by a complex of RNase III-endonucleases Drosha and Dicer to generate pre-miRNAs, and mature miRNAs, respectively [26], [27]. miRNAs cause posttranscriptional repression of protein synthesis by pairing with partially complementary seed sites in the 3′-untranslated regions (UTRs) of target mRNAs, leading to either deadenylation and subsequent mRNA degradation and/or translational inhibition [22], [24], [25], [28]–[31]. Importantly, a single miRNA can regulate expression of hundreds of target genes [32], [33], whereas the expression of a single gene can be regulated by multiple miRNAs [19], [34]. Since their discovery, it has become clear that miRNAs regulate the expression of genes in biological development, differentiation, metabolism, carcinogenesis, immune response and other important cellular and metabolic processes [24], [28], [31], [35]. The functional importance of miRNAs in steroidogenic tissues and cells has not been fully explored; to date limited data exist and that, too, mostly for the ovarian granulosa cells describing the role of miRNAs in the regulation of steroidogenesis-related physiological functions [36]–[42]. Recently, we reported that SR-BI, which delivers the bulk of the cholesterol substrate for steroidogenesis, is regulated by two specific miRNAs, miRNA-125a and miRNA-455, in rat granulosa cells, a model mouse Leydig cell line and the rat adrenal gland [19].

In this study, we performed comprehensive analysis of miRNA profiling using control and *in vivo* hormone treated rat adrenals to identify miRNAs whose expression is altered in response to ACTH, 17α-ethinyl estradiol (17α-E_2_) or dexamethosone (DEX) treatment. Taking cues from the adrenal data, we also examined the effects of Bt_2_cAMP stimulation of rat ovarian granulosa cells and mouse testicular Leydig tumor cells, MLTC-1, on the expression of some of the relevant miRNAs. Furthermore, using a combined *in silico* prediction, quantitative-real-time PCR (qRT-PCR) and Western blot approaches, we also assessed the expression of some predicted target genes. Our results suggest that trophic hormones alter the expression of a number of miRNAs in a cell and hormone specific manner. This information further implicates the potential involvement of miRNAs in the hormonal regulation of steroidogenesis in a posttranscriptional/posttranslational dependent manner.

## Materials and Methods

### Reagents

17α-Ethinyl estradiol (17α-E2), 17β-estradiol (E2), fatty acid poor bovine serum albumin, dexamethasone (DEX) and N^6^,2′-*O*-dibutyryladenosine 3′:5′-cyclic monophosphate (Bt_2_cAMP) were purchased from Sigma Chemical Co. (St. Louis, MO). Collaborative Biomedical Products (Bedford, MA) supplied insulin and transferrin. Cortrosyn® (Cosyntropin; ACTH) was obtained from Amphastar Pharmaceuticals, Inc. (Rancho Cucamonga, CA). Most of the tissue culture supplies were supplied by Life Technologies through its Gibco Cell Culture Media Division (Grand Island, NY). The Pierce® BCA Protein Assay Kit was purchased from Thermo Fisher Scientific, Inc., (Waltham, MA). All other reagents used were of analytical grade.

### Animals and Treatment

All animal experiments were performed according to procedures approved by the VA Palo Alto Health Care System Animal Care and Use Committee (IACUC). Four groups of 225- to 250-g male Sprague Dawley rats (Harlan Laboratories, Indianapolis, IN) were studied (3 animals/group): 1) non-stressed controls (C), 2) rats treated with Cortrosyn (ACTH) (10 IU) every 24 h for 4 days, with the last injection on day 4 given 1 h prior to harvesting of adrenal tissues, 3) rats treated with 17α-E2, 10 mg/kg BW sc every 24 h for 5 days, and 4) rats treated with DEX, a single injection of 100 µg, sc for a 24 h period. At the end of treatment, animals were euthanized and adrenal tissues collected for various molecular and biochemical measurements.

### Isolation, Culture and cAMP Treatment of Rat Granulosa and Mouse Leydig Tumor Cells

Immature female Sprague-Dawley rats (21–23 days old, Harlan Laboratories, Indianapolis, IN) were injected subcutaneously with 17ß-estradiol (1 mg/kg BW sc) daily for 5 days. The animals were euthanized 24 h after their last injection, and granulosa cells were isolated from the ovaries using the follicle puncture method and cultured as described previously [17], [43]. In brief, cells were cultured in 35-mm culture dishes that were precoated with 1% serum. Dishes were plated with 1–2×10^5^ cells in 1.5 ml of basal culture medium [Dulbecco’s modified Eagle’s (DME)-F12 medium supplemented with 15 mM Hepes, bovine serum albumin (1 mg/ml), insulin (2 µg/ml), transferrin (5 µg/ml), hydrocortisone (100 ng/ml), streptomycin (100 µg/ml), and penicillin G (100 U/ml)]. After 72 h of culture, cells were treated with either vehicle alone or Bt_2_cAMP (2.5 mM) for 24 h to cause luteinization of the granulosa cells. Subsequently, the cells were maintained under their respective basal or stimulated (+Bt_2_cAMP) culture conditions for an additional 24 h.

MLTC-1 mouse Leydig tumor cells (catalog no. CRL-2065) were obtained from the American Type Culture Collection (ATCC, Manassas, VA) and cultured in RPMI 1649 medium supplemented with 10% fetal bovine serum. All cell cultures were maintained at 37°C in a humidified incubator containing 5% CO_2_-95% air. When required, cells were incubated with Bt_2_cAMP (2.5 mM) for an appropriate time [17].

### RNA Extraction and Microarray Analysis

Adrenal glands were quickly removed from the euthanized animals and immediately snapped frozen in liquid nitrogen. Samples of these frozen tissues were pulverized and converted to a fine powder using a mortar and pestle which were pre-cooled in liquid nitrogen and subsequently lyzed with the addition of a suitable aliquot of QIAzol Lysis Reagent (QIAGEN Inc., Valencia, CA) and subjected to further grinding. Total RNA from adrenal extracts was isolated using a miRNeasy Mini Kit (QIAGEN) according to the manufacturer’s guidelines. This protocol effectively recovers both mRNA and miRNA. RNA was quantified using Nanodrop (Thomas Fisher Scientific). After assessing RNA quality, miRNA expression profiling was performed using Affymetrix® GeneChip® miRNA 2.0 Arrays, according to the manufacturer’s protocol. The microarray data were analyzed using Partek® Genome Suite software, version 6.3 Copyright© 2008 (Partek Inc., St. Louis, MO, USA). Affymetrix CEL files were processed to generate gcRMA (robust multi-array average) values. The microarray data was submitted to NCBI GEO (GEO accession number: GSE47131). These measurements were carried out by the Protein and Nucleic Acid (PAN) Microarray Facility of Stanford University.

### Quantitative RT-PCR (qRT-PCR) Analysis of mRNA and miRNA Expression

Total RNA was isolated from the whole adrenal gland using a miRNeasy Mini Kit (QIAGEN) as described above. Total RNA was harvested from MLTC-1 and granulosa cells treated without or with Bt_2_cAMP for 6 h or 24 h also using miRNeasy Mini Kit (QIAGEN). To measure mRNA expression, total RNA (2 µg) from cells and adrenals were reverse-transcribed using superscript II reverse transcriptase (Invitrogen, Carlsbad, CA) and oligo(dt) primers. The resulting complementary cDNA samples were used for real-time quantitative PCR using SYBR® Green Real-time Master Mix (Invitrogen) and gene-specific primers (Table 1) on an ABI Prism 7900 HT quantitative PCR system (Applied Biosystems, CA). Target mRNA expression in each sample was normalized to the 36B4 signal. The 2^−ΔΔCt^ method was used to calculate relative mRNA expression levels [49].

**Table 1:** miRNAs that are regulated by ACTH/17α-E2, ACTH/DEX or 17α-E2.

miRNA ID	ACTH 17α-E2(Fold-change)	ACTH DEX(Fold-change)	17α-E2 DEX(Fold-change)
**rno-miR-212**	4.2349 1.7006		
**rno-miR-132**	3.4349 1.6617		
**hp-rno-mir-154**	1.4017 1.4247		
**hp-rno–mir-494**	1.2323 1.5083		
**rno-miR-872**	1.1778 1.1772		
**rno-miR-194**	1.1415 1.1943		
**rno-miR-30a**	1.1363–1.1870		
**hp-rno-mir-24-1**	1.0841–1.0990		
**rno-miR-16**	1.0834 1.1054		−1.1480–1.1450
**hp-rno-miR-322**	−1.1670–1.2340		
**rno-miR-20b-3p**	−1.1970–1.1960		
**rno-miR-339-5p**	−1.2330–1.5180		
**rno-miR-27a**	−1.2700–1.4290	−1.2700–1.3250	−1.4290–1.3250
**rno-miR-551b**	−1.3160–1.3300	−1.3160–1.2930	−1.3300–1.2930
**hp-rno-miR-1224**	−1.4110–1.5060		
**hp-rno-miR-181b-1**		1.3813 1.3435	
**hp-rno-mir-672**		1.1472 1.2173	
**rno-miR-100**		1.0622 1.1142	
**rno-miR-92a**		−1.2190–1.1880	
**rno-miR-466b**		−1.9900–2.3090	
**hp-rno-miR-103-2**			1.2214 1.1799
**hp-rno-miR-320**			1.2077 1.1707
**hp-rno-miR-301a**			1.1633 1.1452
**hp-rno-mir-16**			−1.1480–1.1450
**hp-rno-mir-493**			−1.2390–1.2500
**rno-miR-551b**			−1.3300–1.2930
**hp-rno-mir-122**			−1.3920 1.4020
**rno-miR-200c**			−1.5440–1.4050
**rno-miR-296**			−1.7830–1.6060
**rno-miR-122**			−9.7910–8.2010

To evaluate miRNA expression, the RNA was reverse-transcribed using an miRCURY LNA™ Universal RT microRNA PCR Kit with miRNA-specific primers (MicroRNA LNA™ PCR primer sets, Exiqon, Inc., Woburn, MA). The resulting complementary cDNA samples were used for real-time quantitative PCR using SYBR® Green Real-time Master Mix (Invitrogen) on an ABI Prism 7900 HT quantitative PCR system (Applied Biosystems). Target miRNA expression in each sample was normalized to the small nuclear gene U6 signal. Relative miRNA levels were calculated using the comparative threshold 2^−ΔΔCt^ method [49]. The data are presented as log_2_ of fold change.

### Western Blotting

Adrenals or cells (MLTC-1, granulosa cells) were harvested and homogenized in RIPA buffer (25 mM Tris-HCl, pH 7.6, 150 mM NaCl, 1% NP-40, 1% sodium deoxycholate and 0.1% SDS) supplemented with Thermoscientific Halt™ Protease Inhibitor Cocktail (1 mM AEBSF, 800 nM aprotinin, 50 nM bestatin, 15 nM E-64, 5 mM EDTA, 20 nM leupeptin and 10 nM pepstatin). The lysates were incubated for 20 min on ice and subsequently centrifuged at 10,000 *g* for 5 min. Supernatants were collected, and protein concentrations were determined using the Pierce® BCA Protein Assay Kit. Equal amounts of protein samples (10–20 µg) were separated on SDS-PAGE, transferred to nitrocellulose membranes, and detected using specific antibodies. Membranes were incubated with primary antibodies at the following dilutions: anti-SREBP-1c (1∶500, sc-366; Santa Cruz Biotechnology, Inc., CA), anti-StAR (1∶500, sc-25806; Santa Cruz Biotechnology, Inc., CA), and anti-LDLR(1∶200, homemade), anti-HDAC3 (1∶1000, 2632S; Cell Signaling Biotechnology), anti-SF-1(1∶1000, 07–618; Upstate, Millipore, Billerica, MA), anti-LDLR (1∶1000) and anti-β-Actin (1∶10000, sc-47778; Santa Cruz Biotechnology, Inc., CA, as a loading control for cytosolic and granulosa protein). Ponceau S staining was used as nuclear protein loading control. After three washes with Tris-buffered saline containing 0.1% Tween 20, the membranes were incubated with IRDye® 800CW goat anti-rabbit or IRDye® 6800LT goat anti-mouse secondary antibodies (LI-COR Biosciences) for 1 h. Proteins were detected with the Odyssey® Infrared Imaging System (LI-COR Biosciences).

### Plasmid Construction

The mouse *StAR* sequences–112 bp (206 to 317 nucleotides [nt] from the start of the 3′UTR) and 158 bp (1540 to 1697 nt from the start of the 3′UTR), containing miRNA-138-5p binding site I and miRNA-135-5p binding site II, were amplified by the following sets of primers using oligo (dT) primer/PCR generated cDNAs from MLTC-1 cells as a template: miRNA-138-5p site I, forward primer (F): 5′-CTGAACTAGTGATAAGTAGCTATGAAACC and reverse primer (R): 5′- CTGAAAGCTTGGAGCTGGTAAGACAACAG; miRNA-138-5p site II, forward primer (F): 5′-CTGAACTAGTTTCCAGGAAAGTCAGGGCTG and reverse primer (R): 5′-CTGAAAGCTTCTCTCCTTCCATCTCTGTGG. The mouse *SREBP-1c* sequences, 139 bp (695 to 833 nt from the start of the 3′UTR), containing miRNA-132-3p binding site were amplified by the following sets of primers: forward primer (F): 5′-CTGAACTAGTGAATCTGGTCGGCATCCAC and reverse primer (R): 5′-CTGAAAGCTTCGAGCTGTTTCTAAAAGATG. The mouse *LDLR* sequences, 166 bp (518 to 683 nt from the start of the 3′UTR) containing miRNA-182-5p binding site were amplified by the following sets of primers: forward primer (F): 5′- CTGAACTAGTGTGACTCGTGACATTCGG and reverse primer (R): 5′- CTGAAAGCTTGCAACCAGTTGTCTCAGG. The mouse *LDLR* sequences, 203 bp (292 to 494 nt from the start of the 3′UTR, 173 bp (666 to 838 nt from the start of the 3′UTR) and 137 bp (1066 to 1202 nt from the start of the 3′UTR) containing miRNA-214-3p binding site I, binding site II and binding site III, were amplified by the following sets of primers: miRNA-214-3p site I forward primer (F): 5′- CTGAACTAGTCAAATAGGCTGTCCCAGAAG and reverse primer (R): 5′-CTGAAAGCTTGCAGAGTCAGGCAGGAAG; binding site II forward primer (F): 5′-CTGAACTAGTCCTGAGACAACTGGTTGC and reverse primer (R): 5′-CTGAAAGCTTATGTGGACCACGTGGGTG; binding site III forward primer (F): 5′-CTGAACTAGTCTCCTCTGGTAACGTCATC and reverse primer (R): 5′- CTGAAAGCTTCTCTTCCTCTTGAGCATAGG. Each set of these forward and reverse primers contained HindIII and SpeI recognition sites at the 5′ end of the primers, respectively. The PCR products were digested with HindIII and SpeI and cloned into the 3′ end of the pMIR-REPORT™ Luciferase vector with CMV promoter (Applied Biosystems, Foster City, CA) at HindIII and SpeI sites. The sequences of inserted fragments were confirmed by sequencing.

### Luciferase Reporter Assay

For the luciferase assay, CHO cells at a density of 1×10^5^ per well in 24-well plates were co-transfected with different pMIR-REPORT Luciferase plasmids and different precursor (pre)-miRNAs or scrambled oligonucleotide (Ambion, Life Technologies, Grand Island, NY) using Lipofectamine 2000 reagent (Invitrogen, Carlsbad, CA). Thirty-six hours after transfection, cells were harvested and lysed with passive lysis buffer (Promega, Corp., Madison, WI)). Reporter assays were performed using a dual luciferase reporter assay system (Promega) according to the manufacturer’s instructions. Reporter activity was measured on a SpectraMaxL luminescence microplate reader (Molecular Devices, Sunnyvale, CA). The pRL-TK vector (Promega) *Renilla luciferase* control reporter vector, which provides constitutive expression of *Renilla* luciferase, was used as an internal control. The results are expressed as relative luciferase activities (firefly luciferase/*Renilla* luciferase).

### Bioinformatics Analysis

TargetScan 4.0 and microRNA.org were used to predict target genes for selected miRNAs [50]–[52]. Predicted genes, with microRNA.org scores and TargetScan total context scores exceeding their mean scores, were selected for functional analysis using NCBI and UCSC Genome Browser databases (http://genome.ucsc.edu/).

## Results

### ACTH, 17α-Ethinyl Estradiol and Dexamethasone Regulate miRNA Expression Profiles in Rat Adrenal Gland

**Figure 1:**
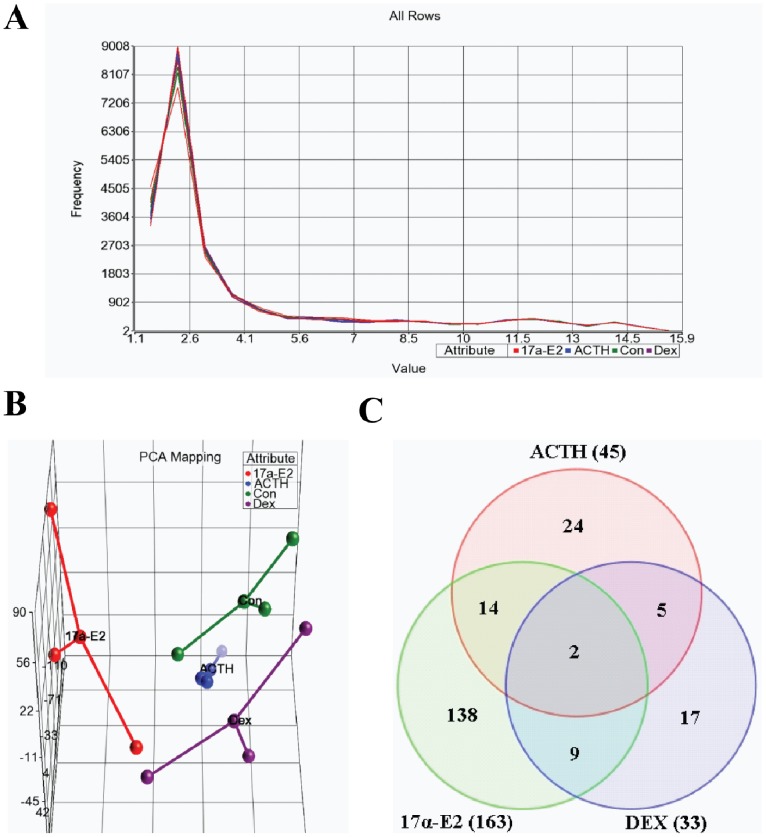
Microarray analysis of miRNAs in control and ACTH, 17α-E2 and DEX treated rat adrenals. [A]. Frequency of expressed values. These data show the reproducibility and consistancy among all the samples. [B]. 3-D View of Principal Component Analysis (PCA) showing distinguished clusters between control and ACTH, 17α-E2 and DEX treated adrenals. The PCA was performed on differentially expressed genes between control and ACTH, 17α-E2 and DEX treated adrenals. Adrenals without or with ACTH, 17α-E2 or DEX are represented by different colors i.e., green for control, blue for ACTH, red for 17α-E2 and purple for DEX. The control, ACTH, 17α-E2 and DEX treated adrenals clustered into different and distinct groups. [C]. Venn diagram representing differentially expressed miRNAs observed in the comparisons among the adrenals treated with ACTH, 17α-E2 or DEX.

To assess the impact of hormonal treatment *in vivo* on changes in miRNA profiles, groups of three rats were treated with normal saline (control), ACTH, 17α-ethinyl estradiol (17α-E2) or dexamethasone (DEX) as described in the experimental section. Previous studies have shown that chronic ACTH treatment leads to up-regulation of steroidogenic machinery [44] and causes enhanced expression of adrenal low-density lipoprotein receptor (LDL-R) [45] and scavenger receptor class B, type I (SR-BI) [46]. Treatment of rats with 17α-E2, a potent hypocholesterolemic agent [46]–[48], also leads to induction of both adrenal LDL-R (the current study) and SR-BI [46]. DEX treatment inhibits the adrenal HPA axis [53] and pituitary ACTH secretion [44], [53] and, as a result, downregulates the expression of adrenal LDL-R [44], SR-BI [45] and plasma corticosterone levels [46]. Total RNA isolated from 2 adrenals of three individual animals treated with vehicle, ACTH, 17α-E2 or DEX was applied to Affymetrix® GeneChip® miRNA 2.0 Arrays containing 131 organisms 20706 probes (including mature miRNA, pre-miRNA transcript, sno/scaRNAs and Affymetrix control sequence). Among the total 20706 signatures, there are 772 rat related signatures. Evaluation of the frequency of the normalized expression values of the data showed reproducibility and consistency among the samples (**Fig. 1A**). Principal component analysis revealed a clear distinction between the treatment groups. Generation of a non-censored PCA plot using all miRNAs showed that samples with different treatment clustered into different distinct groups (**Fig. 1B**). This clustering represents the overall expression patterns, but does not provide information about the expression of individual genes. The Venn diagram (**Fig. 1C**) summarizes the number of differentially expressed miRNAs in the adrenals from animals treated with ACTH, 17α-E2 or DEX.

**Figure 2:**
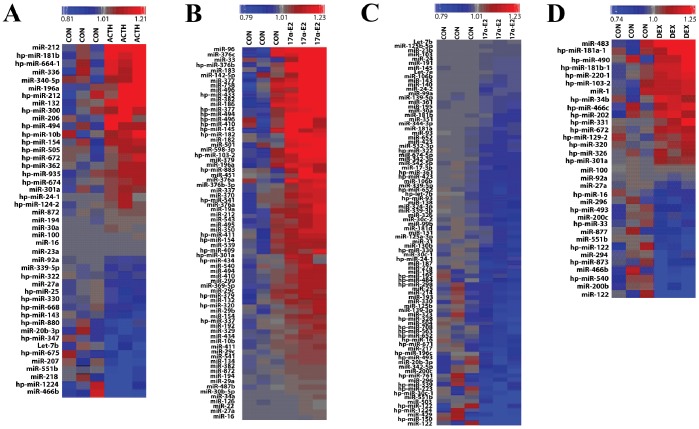
MicroRNA (miRNA) expression profiles in adrenals from rats treated with ACTH, 17α-E2, DEX or saline (control). [A]. The heat map represents the expression levels of 45 miRNAs in two conditions (control and ACTH). [B, C]. The heat map represents the expression levels of 163 miRNAs in two conditions (control and 17α-E2). 74 miRNAs were up-regulated [B] and 89 miRNAs were down-regulated with 17α-E2 [C]. [D]. The heat map represents the expression levels of 33 miRNAs in two conditions (control and DEX). Red, up-regulated genes; blue, down-regulated genes.

**Figure 3:**
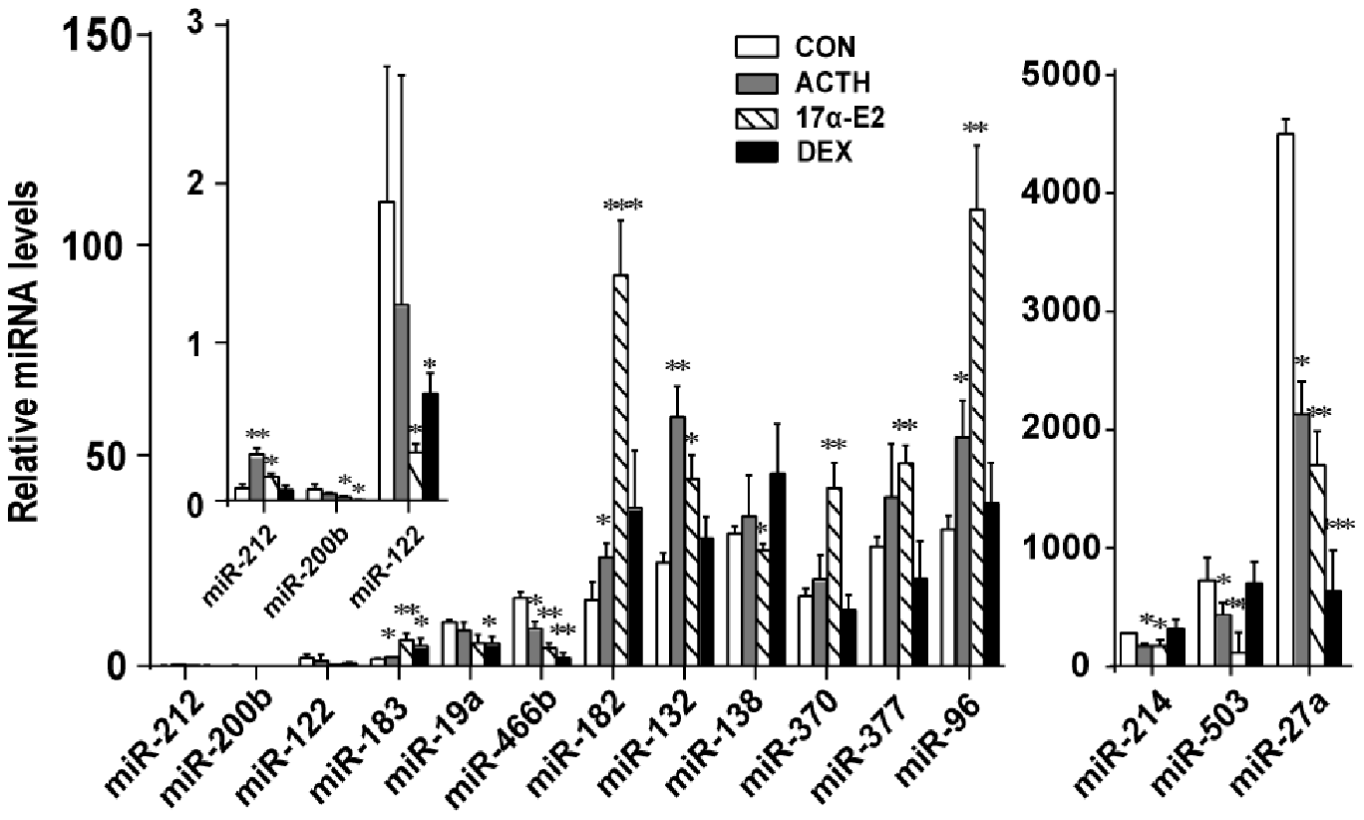
Quantitative RT-PCR (qRT-PCR) validation of miRNA-212, miRNA-200b, miRNA-183, miRNA-122, miRNA-19a, miRNA-466b, miRNA-182, miRNA-132, miRNA-138, miRNA-370, miRNA-96, miRNA-503, miRNA-27a and miRNA-214 levels in control, ACTH-, 17α-E2 or DEX-treated adrenals *in vivo*. Expression of U6 was used for normalization. The experiments were performed independently three times. Data are presented as mean ± standard error. *p<0.05; **p<0.01; ***p<0.001.

Using Partek Genomics Suite, 45 out of the 772 expressed rat miRNAs showed significant changes (p<0.05) in the adrenal in response to ACTH treatment. Among these, 27 mature or precursor miRNAs were up-regulated and 18 mature or precursor miRNAs were down-regulated in ACTH-exposed adrenals versus control adrenals; *p*<0.05 (**Fig. 2A**). ACTH treatment caused maximum up-regulation of two miRNAs, miRNA-212 and miRNA-132, with a fold-stimulation of 4.23 and 3.43, respectively. The precursor for miRNA-212 was also up-regulated. Real-time PCR (qRT-PCR) confirmed ACTH-mediated up-regulation of miRNA-212, miRNA-183, miRNA-182, miRNA-132 and miRNA-96. Significant ACTH-induced down-regulation of miRNA-466b, miRNA-214, miRNA-503 and miRNA-27a was also observed (**Fig. 3**). While this work was in progress, a recent microarray study reported the expression profile of miRNAs in mouse adrenals in response to acute treatment of animals with ACTH and demonstrated no similarities with our observations [41].

We also performed a microarray analysis to screen the expression profiles of miRNAs in adrenals from rats chronically treated with a hypocholesterolemic and possible ACTH secretagogue, 17α-E2 [45]–[47]. The expression levels of 163 mature miRNAs varied significantly (*p*<0.05) in response to 17α-E2 treatment (**Fig. 2B, C**), of which, 63 miRNAs exhibited changes more-than 1.5 fold (p<0.05). The expression levels of miR-183 (4.61-fold), miR-96 (4.56-fold), and miR-182 (4.29-fold) were most highly up-regulated, whereas miR-122 (9.79-fold), miR-503 (5.88-fold), and miR-139-3p (1.94-fold) showed the greatest down-regulation as a result of 17α-E2 treatment. qRT-PCR measurements confirmed that the expression of miR-212, miRNA-183, miRNA-182, miRNA-132, miRNA-370, miRNA-377 and miRNA-96 was up-regulated and that of miRNA-122, miRNA-200b, miRNA-466b, miRNA-138, miRNA-214, miRNA-503 and miRNA-27a down-regulated in adrenals from 17α-E2 treated rats (**Fig. 3**).

To identify a set of adrenal miRNAs that are potentially regulated by a potent synthetic glucocorticoid agonist and an inhibitor of corticosteroidogenesis, dexamethasone [45], three rats were treated with a single dose of dexamethasone (100 µg) sc for a 24 h period. Again, changes in the adrenal miRNA profile on dexamethasone treatment were assessed using microarray. Analysis of the miRNA array showed that expression of 33 miRNAs significantly changed (p<0.05) following treatment with dexamethasone (**Fig. 2D**). The expression of miRNA-483, miRNA-181a-1, miRNA-490 and miRNA and miRNA-181b-1 was up-regulated in response to dexamethasone treatment. In contrast, dexamethasone down-regulated the expression of several of the miRNAs by more than 1.5 fold, i.e., miR-122 (8.2-fold), miR-466b (2.31-fold), miR-200b (1.9-fold) miR-877 (1.61-fold), miR-296 (1.61-fold)and precursor of miR-504 (1.53-fold) (**Fig. 2D**). Expression of miRNA-27a (**1.32**-fold) was also down-regulated by DEX. Using qRT-PCR, we confirmed the down-regulation of miRNA-200b, miR-122, miR-19a, miRNA-466b, and miRNA-27a expression (**Fig. 3**).

### Multiple Hormonal Regulation of Adrenal miRNAs

We also examined whether the expression of any miRNAs is altered by more than one hormone treatment, i.e., by ACTH/17α-E2, ACTH/DEX, 17α-E2/DEX or ACTH/17α-E2/DEX. The results are presented in **Table 1**. The level of expression of miR-212 and miR-132 was up-regulated (>1.5-fold) by both ACTH and 17α-E2 treatments. ACTH and DEX down-regulated miR-466b more than 1.5-fold, but the effect of 17α-E2, although it showed a similar trend, was not statistically significant (p = 0.084). miR-296 and miR-122 were down-regulated (>1.5 fold) by both 17α-E2 and DEX. The levels of miR-27a and miR-551b were down-regulated by all three hormones, ACTH, 17α-E2 and DEX. Microarray data demonstrated that the levels of miR-183 and miR-182 were up-regulated with 17α-E2 treatment, but not with ACTH (*p* = 0.065) treatment; qRT-PCR measurements, however, showed significant increases in their expression in response to either ACTH or 17α-E2 treatment.

### cAMP Induced Regulation of miRNA Expression in Primary Rat Granulosa Cells and Mouse Leydig Tumor MLTC-1 Cells

**Figure 4:**
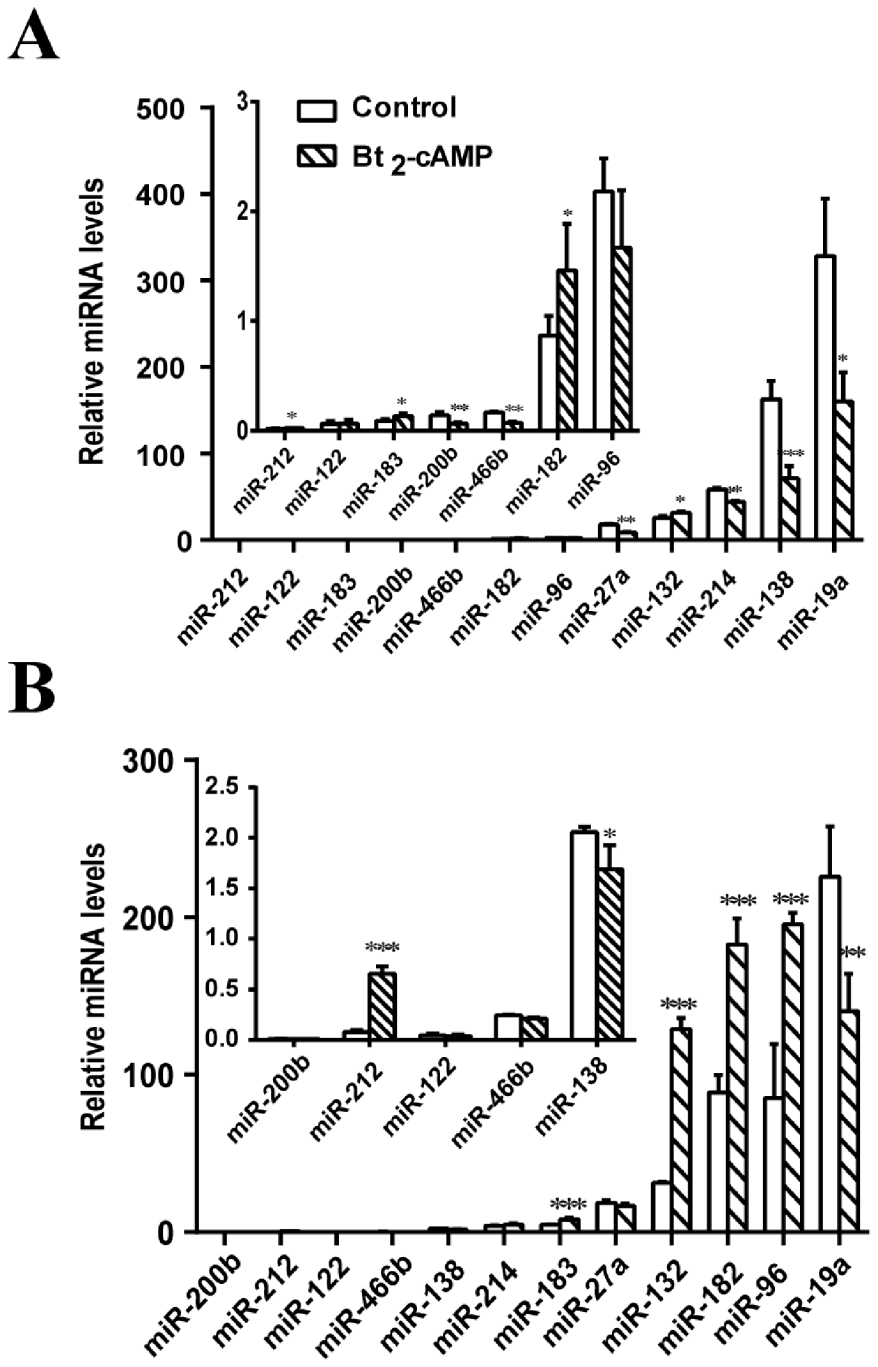
Quantitative RT-PCR (qRT-PCR) analysis of miRNAs in mouse rat granulosa and MLTC-1 cells treated without or with Bt_2_cAMP (2.5 mM) for 24 h or 6 h. [A] Granulosa cells: groups of RNA samples were analyzed by qRT-PCR. The levels of expression of miRNA-212, miRNA-122, miRNA-138, miRNA-214, miRNA-183, miRNA-182, miRNA-132, miRNA-96, miRNA-466b, miRNA-200b, and miRNA-19a are shown. Expression of U6 was used for normalization. [B] MLTC-1 cells: groups of RNA samples were analyzed by qRT-PCR. The levels of expression of miRNA-212, miRNA-122, miRNA-138, miRNA-214, miRNA-183, miRNA-182, miRNA-132, miRNA-96, miRNA-466b, miRNA-200b, and miRNA-19a are shown. Expression of U6 was used for normalization. *p<0.05; **p<0.01; ***p<0.001.

We next examined whether the expression of some of the miRNAs that were found to be hormone-sensitive in the adrenal were also regulated in granulosa and MLTC-1 cells treated with a cAMP agonist, Bt_2_cAMP. More specifically, we assessed the impact of Bt_2_cAMP treatment on the expression of miRNA-212, miRNA-122, miRNA-27a, miRNA-466b, miRNA-200b, miRNA-138, miRNA-214, miRNA-183, miRNA-182, miRNA-132, miRNA-96 and miRNA-19a. qRT-PCR measurements indicated that exposure of primary rat granulosa cells to Bt_2_cAMP for 24 h inhibited the expression of miRNA-200b, miRNA-466b, miRNA-27a, miRNA-214, and miRNA-138 and miRNA-19a while enhancing the expression of miRNA-212, miRNA-183, miRNA-182, and miRNA-132 (**Fig. 4**). Treatment of MLTC-1 cells with Bt_2_cAMP for 6 h increased the expression of miRNA-212, miRNA-183, miRNA-132, miRNA-182 and miRNA-96 and inhibited the expression of miRNA-138 and miRNA-19a (**Fig. 4B**).

### Correlation of Expression Levels of Selected miRNAs with their Predicted Targeted Genes

Having identified miRNAs that are subject to hormonal regulation, we next examined potential correlations between selected hormone-sensitive miRNAs with their predicted target genes. We first used microRNA.org and TargetScan 4.0 to predict target genes for selected hormone-responsive miRNAs. Some miRNAs down-regulate large numbers of target mRNAs through interaction with 3′ UTRs (Lim et al., 2005). The number of target genes predicted by a single miRNA varied greatly, ranging from several to hundreds. Using NCBI databases for functional screening of the putative target genes, we further identified a number of target genes directly involved in steroidogenesis (**Table 2**). We also performed a reverse prediction strategy, based on the sequence of the 3′ UTRs of the gene of interest, to make a prediction about the miRNAs which may target some critical steroidogenic genes, such as CYP11A1, StAR, LDL-R and NR5A1. CYP11A1, the gene encoding cholesterol side-chain cleavage enzyme (P450scc), was predicted to be the target gene of miRNA-134. StAR may be a target gene of miR-376b, miR-150, miR-330 and miR-138. NR5A1 was predicted to be the target gene of miR-342, while LDL-R was predicted to be the target gene of miR-182 and miR-466b. MiR-183, miR-96 and miR-19a were predicted to target the ABCA1 gene. ABCG1 may be a target gene of miR-542.

**Table 2:** miRNAs and gene targets.

miRNA	Chromosomallocation (rat)	Regulation	Predicted Target Genes
**miR-212/−132**	10	AU, EU	SOX5, FOXA1, FOXO3,CREB5, ABCG4, RICS, SREBP-1c, GPAT2, MECP2
**miR-154**	6	AU, EU	DOCK1, MECP2, WNT5A
**miR-183**	4	AU, EU, DU	FOXO1, ABCA1, NR3C1
**miR-182**	4	AU, EU	FOXO3, IGF1R,, ABCD1, CREB1, SOX6, CEBPA, LDL-R, SIK1, PIK3R1, FOXO1
**miR-207**	5	AD	CHREBP, LEPTIN
**miR-218**	14	AD	SNF1LK2, PIK3R1, ABCG4, SOX5, MECP2
**miR-450a**	X	AD	CREB1, IGF1
**miR-96**	4	EU	SOX5, ABCD1, FOXO1, FOXQ1, VLDLR, IGF1R, ABCA1, FOXO4, SNF1LK, ABCA2, FOXO3, PIK3R1, MECP2
**miR-494**	6	EU, AU	PTEN, ROCK1, IGF1R
**miR-376b**	6	EU, DD	CTBP2, IGF1R, STAR
**miR-377**	6	EU	NR6A1, SMAD4, WNT5A
**miR-32**	5	EU	PTEN, GATA2, NR4A3, SNF1LK, ABCG4
**miR-150**	1	ED	LXRB, STAR, ACBD3
**miR-504**	X	ED, AD	CYP11B1, HSD17B7, HSD17B8
**miR-370**	6	EU	FOXO3
**miR-19a**	15	EU	IGF2R, MECP2, PPARA, PIK3R3, FOXP2, PTEN, ABCA1, FOXP1, WNT1, SMAD4, SOX5, IGF1R
**miR-138**	8	AD, ED	PPARD, PPARGC1A, SNAP25, STAR
**miR-125b-2**	11	ED, AD	CYP24A1
**miR-503**	X	AD, ED	CYP26B1, SNF1LK, IGF1R, WNT3A, SOX5, PAPPA, WNT4
**miR-122**	18	ED, DD	GATA4, FOXO3
**miR-99b**	1	ED	IGF1R, CYP26B1
**miR-466b**	17	AD, DD	IGF1R, LDLR, SREBP1
**miR-483**	1	DU	IGF1, SMAD4
**miR-200b**	5	DD	NR5A2, CREB5, SNAP25, GATA4, SNF1LK2, CYP1B1, SOX2, PPARA, SOX1, PTEN, MECP2, FOXO3
**miR-881**	X	DD	PDK2
**miR-342**	6	ED	SOX6, NR5A1
**miR-330**	1	AD	MECP2, STAR
**miR-27a**	19	AD, ED, DD	SNAP25, PPARG, NR5A2, SMAD5, CREB1, ABCA1, CYP39A1, GATA2, PDK1, WNT3A, FOXO1, CYP1B1, PPARA, GSK3B, IGF1
**miR-542**	X	ED	CYP17A1, LXRB, HSD17B11, ABCG1, ABCA2, IGF2
**miR-350**	13	EU	GATA3, PIK3R3, CYP26B1, SNAP25, DAX1, FOXA2, CTBP1, IGF1R, HOXA1, CREB5, SOX5, FOXO3
**miR-134**	6	EU	CYP11A1
**miR-214**	13	AD, ED	VLDLR, WNT3, PPARGC1a, FOXO4, LDLR, CREB1, SCARB1, UCP2
**miR-1**	18	AU, EU, DU	IGF1, CREB5, SNAP25, NR4A2, SMAD4

AU, ACTH up-regulated; EU, 17α-E2 up-regulated; DU, DEX up-regulated; AD, ACTH down-regulated; ED, 17α-E2 down-regulated; DD, DEX down-regulated.

**Figure 5:**
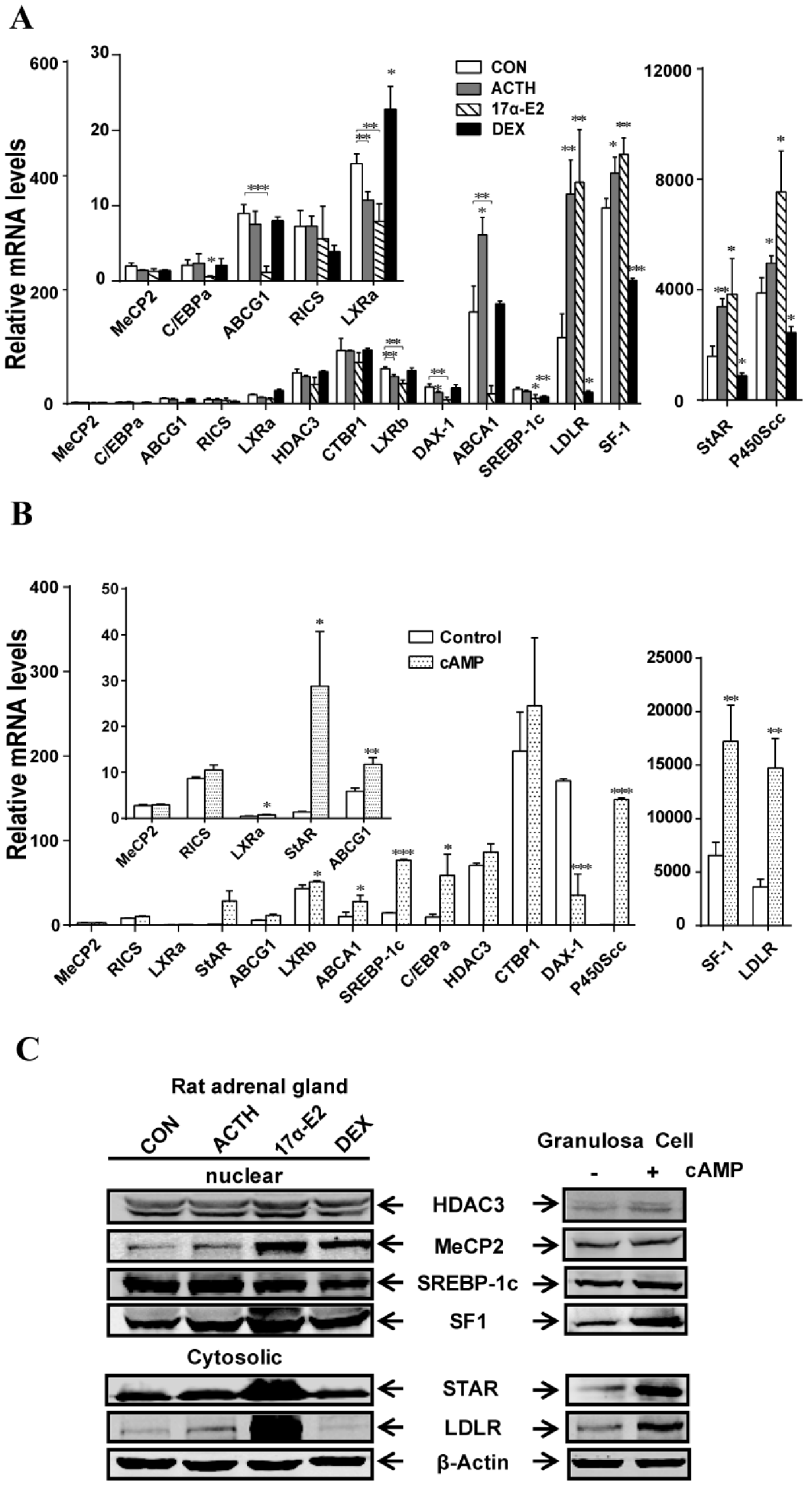
Quantitative RT-PCR (qRT-PCR) and Western blot analysis of several putative miRNAs target genes related to steroidogenesis. (A) qRT-PCR analyses of selected genes in rat adrenal glands regulated by ACTH, 17α-E2 and DEX. (B) qRT-PCR analyses of Bt_2_cAMP regulated genes in rat granulosa cells. 36B4 was used for normalization. *p<0.05; **p<0.01; ***p<0.001. C. Western blot analysis of SREBP-1c, SF-1, HDAC3, StAR, LDLR and β-actin in different treated rat adrenals and rat granulosa cells.

In a follow-up study, we performed real-time PCR and Western blot analysis to monitor expression of predicted target genes and their protein products in response to hormone treatment of rat adrenals and ovarian granulosa cells. Three genes, *Mecp2, Ctbp1 and p250 GAP,* have been recently identified as targets of miR-132 [36]. However, in our RT-PCR assay adrenal mRNA levels of *Mecp2*, *Ctbp1* and *Rics* were not impacted by ACTH, DEX or 17α-E2 treatment. Likewise, expression of another predicted target gene of miR-132, HDAC3, was also unchanged by ACTH, 17α-E2 or DEX treatment (Fig. 5A).

As summarized in **Table 2**, several genes involved in lipid metabolism and steroidogenesis were predicted to be the target genes of different miRNAs. We examined the expression of some of these predicted lipid/steroidogenic genes in the adrenals from control and hormone treated rats. qRT-PCR data showed that mRNA levels of SF-1, StAR, CYP11A1 and LDL-R were all up-regulated in the rat adrenal gland in response to ACTH and 17α-E2, but down-regulated with DEX treatment (**Fig. 5A**). Both ACTH and 17α-E2 treatment of rats caused a significant reduction in mRNA levels of LXRα, LXRß, and DAX-1. Interestingly, mRNA levels of LXRα were up-regulated by DEX treatment. Adrenal mRNA levels of ABCA1, ABCG1 and C/EBPα were significantly reduced in 17α-E2 treated animals, while ACTH treatment increased the mRNA expression of ABCA1. Finally, the mRNA expression of SREBP-1c was significantly attenuated in response to 17α-E2 or DEX treatment.

We also determined the mRNA levels of these genes in rat granulosa cells treated with or without Bt_2_cAMP (**Fig. 5B**). In rat granulosa cells, the mRNA levels of *Rics*, *Ctbp1*, HDAC3 and MECP2 were not affected following treatment with Bt_2_cAMP. SF-1, StAR, CYP11A1, LDL-R, LXRα, LXRß, ABCA1, ABCG1, SREBP-1c, and C/EBPα mRNA levels were all up-regulated in cAMP-treated rat granulosa cells, whereas mRNA levels of DAX-1 transcription factor were significantly reduced. Using Western blot, we also assessed the effects of hormonal treatment of rat adrenal gland and Bt_2_cAMP treatment of rat granulosa cells on the levels of StAR, SF-1, SREBP-1c, LDLR and HDAC3 proteins, and the results are presented in **Fig. 5C**. Western blot analysis showed no significant changes in the nuclear protein levels of HDAC3 in ACTH, 17α-E2 or DAX treated adrenals. The adrenal protein levels of StAR and SF-1 were increased significantly following treatment of rats with ACTH or 17α-E2, although DEX treatment showed no significant effect on the protein levels of either of these two proteins. The protein levels of lipogenic transcription factor, SREBP-1c, were decreased in the 17α-E2, as well as DEX, treated adrenals. In contrast and as expected, a significant increase in LDL-R protein levels was noted in the 17α-E2 treated adrenal samples. In ovarian granulosa cells, protein levels of StAR, SF-1, SREBP-1c and LDL-R were all increased following Bt_2_cAMP treatment. As before, HDAC3 levels, however, were not impacted by Bt_2_cAMP stimulation.

### miRNA-132 and miRNA-214 Suppress SREBP-1c and LDLR by Targeting Specific Site(s) within the 3′ UTR of SREBP-1c and LDLR, Respectively

**Figure 6:**
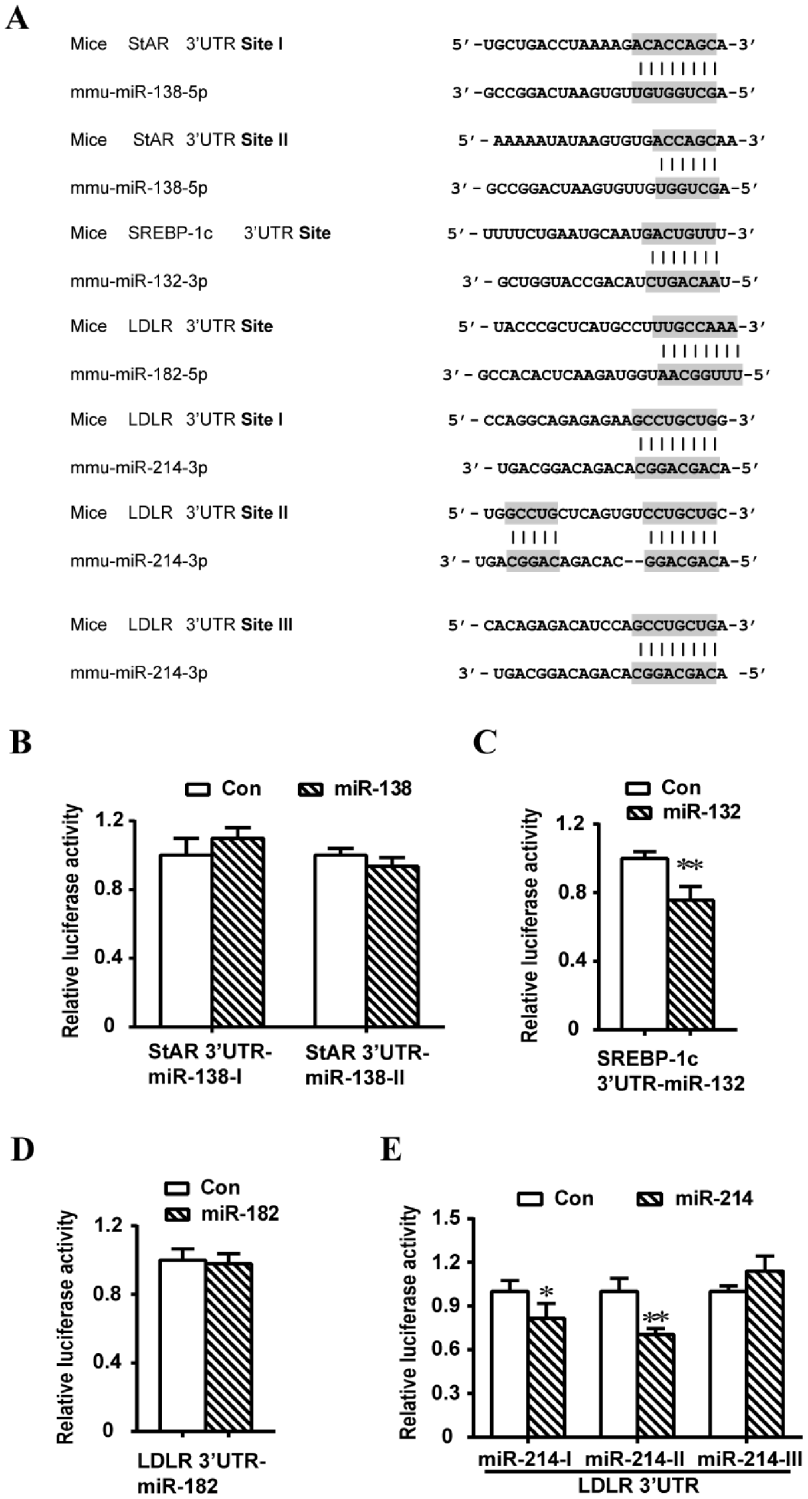
miRNA-132 and miRNA-214 binding sites in the 3′ UTR of the mouse *SREBP-1c* and *LDLR* genes mediate the downregulation of SREBP-1c and LDLR expression by miRNA-132 and miRNA-214, respectively. [A]. Seed sequences of the putative miRNA-138-5p, miRNA-132-3p and miRNA-182-5p/miRNA-214-3p binding sites in the 3′-UTR of mouse *StAR*, *SREBP-1c* and *LDLR* genes, respectively. For the reporter gene assay, the 3′ UTR region of the StAR gene containing site I or site II binding site for miRNA-138-5p, the 3′-UTR of SREBP-1c containing a binding site for miRNA-132-5p, the 3′-UTR of LDLR containing a binding site for miRNA-182-5p or the 3′-UTR of LDLR containing site I, site II, or site III binding site for miRNA-214-3p was inserted downstream of the luciferase open reading frame of pMIR-REPORT vector. CHO cells were co-transfected individually with the StAR 3′-UTR (containing putative site I or site II for miRNA-138 binding) ± pre-miRNA-138-5p (panel B), the SREBP-1c 3′-UTR (containing putative binding site for miRNA-132) ± pre-miRNA-132-3p (panel C), the LDLR 3′-UTR (containing putative binding site for miRNA-182) (panel D), or the LDLR 3′-UTR (containing putative site I, site II or site III for miRNA-214 binding) ± pre-miRNA-214-3p for 36 h (panel E). Reporter gene assays were performed using a dual-luciferase kit as described in Materials and Methods. The results are expressed as relative luciferase activities (firefly luciferase/*Renilla* luciferase).

The results presented above raised the possibility that some of the hormone sensitive miRNAs might posttranscriptionally/translationally alter the expression of certain proteins involved in lipid metabolism. Here, we directly assessed the binding of miRNA-138, miRNA-132 and miRNA-182/miRNA-214 to the 3′UTR of StAR, SREBP-1c, and LDLR, respectively, and regulation of their expression levels, by carrying out luciferase reporter gene assays. Individual fragments of the 3′ UTR region of the StAR gene containing site I or site II binding site for miRNA-138-5p, the 3′-UTR of SREBP-1c containing a binding site for miRNA-132-5p, the 3′-UTR of LDLR containing a binding site for miRNA-182-5p or the 3′-UTR of LDLR containing site I, site II, or site III binding site for miRNA-214-3p were inserted downstream of the luciferase open reading frame of pMIR-REPORT vector. CHO cells were co-transfected individually with StAR 3′-UTR (containing the putative site I or site II for miRNA-138 binding) ± pre-miRNA-138-5p (panel B), SREBP-1c 3′-UTR (containing the putative binding site for miRNA-132) ± pre-miRNA-132-3p, LDLR 3′-UTR (containing the putative binding site for miRNA-182), or LDLR 3′-UTR (containing the putative site I, site II or site III for miRNA-214 binding) ± pre-miRNA-214-3p for 36h, followed by determination of luciferase activities. Overexpression of pre-miRNA-132 and pre-miRNA-214 significantly decreased the luciferase activity of the 3′UTR of the SREBP-1c and LDLR reporter containing micRNA-132 and miRNA-214 binding sites, respectively (**Fig. 6**). In contrast, no inhibitory effect of pre-miRNA-138 on the StAR 3′ UTR (with 2 putative binding sites) reporter construct and pre-miRNA-182 on the LDLR 3′UTR (with a single putative binding site) reporter construct was detected.

## Discussion

Steroid hormone synthesis occurs predominantly in the steroidogenic cells of the adrenal gland, ovary and testis and is under the control of trophic peptide hormones secreted from the pituitary. The rate limiting step in steroidogenesis is the trophic hormone−/cAMP-stimulated and StAR-mediated translocation of cholesterol from the outer mitochondrial membrane to the inner mitochondrial membrane where the side-chain cleavage enzyme (P450scc; Cyp11A1) carries out the first committed step in steroidogenesis, i.e., conversion of cholesterol to pregnenolone [1]–[4]. This step is subject to both acute [3], [5]–[8] and chronic [2], [3], [9]–[12] stimulation, and trophic hormones regulate this step mainly at the level of gene transcription. Although limited information is also available to suggest that posttranscriptional and posttranslational events may be involved in the regulation of steroidogenesis, relatively little information is available on the biological factors that possibly mediate these events. Emerging evidence showing hormonal regulation of miRNAs in steroidogenic cells [19], [36]–[42], coupled with the identification of a diverse and large number of miRNAs [21]–[25], strongly suggest that miRNAs may be involved in the posttranscriptional/posttranslational regulation of steroidogenesis. In this study, we first carried out a comprehensive analysis of miRNA profiling using control and *in vivo* hormone treated rat adrenals to identify miRNAs whose expression is altered in response to ACTH, 17α-ethinyl estradiol (17α-E_2_) or dexamethosone (DEX) treatment. Taking cues from the adrenal data, we also examined the effects of Bt_2_cAMP (the second messenger of trophic hormone action) stimulation of rat ovarian granulosa cells and mouse testicular Leydig tumor cells, MLTC-1, on the expression of some of the relevant miRNAs.

Chronic ACTH treatment *in vivo* significantly altered the levels of many miRNAs in rat adrenal glands. In general, more miRNAs were upregulated than downregulated in response to ACTH treatment. Real-time PCR (qRT-PCR) measurements demonstrated that ACTH treatment upregulated the expression of miRNA-212, miRNA-183, miRNA-182, miRNA-132 and miRNA-96, while down-regulating the expression of miRNA-466b, miRNA-214, miRNA-503 and miRNA-27a. However, the levels of expression of these miRNAs differed considerably when measured by real-time-PCR as compared to their expression values detected by microarray analysis. This result is most likely due to the detection of both precursor and mature forms of miRNAs by microarray, and only the mature form by PCR [54]. While our work was in progress, a microarray study reported the expression profile of mouse adrenal miRNAs under basal conditions (0 time) and in response to acute treatment of mice (10, 30 or 60 min) with ACTH [41]. In that study, 16 miRNAs were identified, whose levels of expression were maximally up-regulated following 10 min treatment of mice with ACTH (range: 1.1180–1.8437), whereas expression of one miRNA, mmu-mRNA-433, was down-regulated (–1.1465). Those miRNAs differentially expressed on the microarrays with greatest fold changes, miRNA-101a, miRNA-142-3p, miRNA-433 and miRNA-96, were further analyzed. Both microarray and qRT-PCR data measurements indicated that the expression of these four miRNAs varied considerably with respect to ACTH treatment and time after treatment [41]. Moreover, significant differences were also noted between microarray and qRT-PCR measurements. Interestingly, a comparison of our gene array list to the list presented in this publication [41] indicates that none of the transcripts overlap. The reasons for this observed disparity are not clear, but may stem from many factors, including the use of two different types of rodent adrenals (mouse vs rat) and two different ACTH treatment regimens (chronic vs acute ACTH treatment).

In addition to ACTH, we also performed a microarray analysis to screen the expression profiles of adrenal miRNAs from rats chronically treated with 17α-E2, a hypocholesterolemic and possible ACTH secretagogue [46]–[48]. 17α-E2 treatment, like ACTH treatment, results in the induction of both adrenal LDL-R (the current study) and SR-BI [46]. To our knowledge, this is a first report describing the effects of 17α-E2 on the expression of adrenal miRNAs. Significant differences in expression of 163 miRNAs were observed between the adrenals from 17α-E2-treated rats and control rats, with 63 miRNAs showing a change greater than 1.5-fold. The expression levels of miR-183, miR-96, and miR-182 were most highly up-regulated, whereas miR-122, miR-503, and miR-139-3p exhibited the greatest down-regulation as a result of 17α-E2 treatment. Real-time quantitative PCR measurements confirmed that the expression of miR-212, miRNA-183, miRNA-182, miRNA-132, miRNA-370, miRNA-377 and miRNA-96 was up-regulated and that of miRNA-122, miRNA-200b, miRNA-466b, miRNA-138, miRNA-214, miRNA-503 and miRNA-27a down-regulated in adrenals from 17α-E2 treated rats. Again, as noted above for ACTH treatment, the expression levels of miRNAs differed significantly between measurements made by microarray analysis and qRT-PCR. Furthermore, a comparison of ACTH data with that of 17α-E2 data demonstrated that only ∼25% of the transcripts overlap. This suggests that 17α-E2-induced hypocholesterolemia or direct estrogen effects on the adrenal, but not increased ACTH secretion, is most likely responsible for the observed alterations in the levels of specific miRNAs in adrenals of 17α-E2-treated rats.

The hypothalamus-pituitary-adrenal (HPA) axis consists of a set of direct influences and feedback responses between the hypothalamus, the pituitary gland and the adrenal that control reactions to stress and glucocorticoid secretion. Glucocorticoid (cortisol in humans and corticosterone in rodents) secretion by the adrenal cortex inhibits the functions of both the hypothalamus and the pituitary gland by a negative feedback mechanism. This reduces the secretion of CRH and vasopressin and directly reduces the cleavage of pro-opiomelanocortin (POMC) into ACTH and ß-endorphin. In our study, we examined the impact of a synthetic glucocorticoid, dexamethasone (DEX)-mediated inhibition of the HPA axis and ACTH secretion, on miRNA expression profiles in the adrenals [53]. DEX treatment up-regulated the expression of miRNA-483, miRNA-181a-1, miRNA-490 and miRNA-181b-1, while it down-regulated the levels of miR-122, miR-466b, miR-200b, miR-877, miR-296, miRNA-27a and precursor of miR-504. Furthermore, such DEX alteration of adrenal miRNA levels demonstrates that DEX suppression of endogenous ACTH secretion modulates a set of adrenal miRNAs, with the exception of miRNA-96, miRNA-466, and miRNA-27a, that are distinct from those modulated by treatment with exogenous ACTH. Interestingly, the expression of miRNA-96 is up-regulated in response to ACTH treatment, but is down-regulated following DEX treatment. Considering the current view that miRNAs act as negative regulators of gene expression, their altered expression in response to DEX may enhance and/or reduce the expression of target steroidogenic genes, leading to possibly down-regulation of adrenal steroid hormone synthesis and secretion.

Our data further demonstrate that expression levels of some miRNAs are regulated by more than one hormone, i.e., by ACTH/17α-E2, ACTH/DEX, 17α-E2/DEX or ACTH/17α-E2/DEX; **Table 1**. The most striking similarity was observed between ACTH and 17α-E2. Both ACTH and 17α-E2 up-regulated the expression of miRNA-212, miRNA-132, miRNA-154, miRNA-494, miRNA-872, miRNA-194, and miRNA-24-1, but reduced the expression of miRNA-322, miRNA-20b, miRNA-339, miRNA-27a, miRNA-551b, and miRNA-1224. We also observed that miRNA-30a was up-regulated in adrenals treated with ACTH, but down-regulated by 17α-E2 exposure. A comparison of effects of ACTH and DEX shows that both hormones increased the expression miRNA-181b, miRNA-672, and miRNA-100, and significantly decreased the levels of miRNA-92a, and miRNA-466b. In addition to ACTH/17α-E2 and ACTH/DEX, we observed that a total of 11 miRNAs are regulated by both 17α-E2 and DEX. Among these, three mRNAs were up-regulated in response to *in vivo* treatment of adrenals with 17α-E2 or DEX, and the remaining eight miRNAs were down-regulated in treated adrenals with either of the two hormones. Finally the expression levels of miRNA-27a and miRNA-551b were significantly reduced in adrenals of ACTH, 17α-E2 or DEX treated animals. Together, these data raise the possibility that some of these miRNAs (with sensitivity towards two or three hormones) may be intimately involved in the complex regulation of adrenal steroidogenesis.

We next evaluated the effects of Bt_2_cAMP stimulation of rat ovarian granulosa cells and of mouse MLTC-1 Leydig tumor cells on the expression of twelve miRNAs (miRNA-212, miRNA-122, miRNA-183, miRNA-200b, miRNA-466b, miRNA-182, miRNA-96, miRNA-27a, miRNA-132, miRNA-214, miRNA-138 and miRNA-19a) whose adrenal expression was differentially altered in response to treatment of rats with ACTH, 17α-E2 or DEX. qRT-PCR measurements indicated that in granulosa cells, miRNA-138 and miRNA-19a are expressed at very high levels as compared to other miRNAs. Significant expression was also observed for miRNA-27a, miRNA-132 and miRNA-214, whereas very low expression was noted for all of the remaining (seven) miRNAs. Bt_2_cAMP stimulation of granulosa cells caused down-regulation of a majority of miRNAs, including miRNA-200b, miRNA-466b, miRNA-27a, miRNA-214, miRNA-138 and miRNA-19a, but expression levels of miRNA-212, miRNA-183, miRNA-182, and miRNA-132 were significantly increased. The expression levels of miRNA-122 and miRNA-96, however, were not affected by cAMP stimulation. A few earlier studies have examined the expression of miRNAs, although these studies were mainly focused on identifying miRNAs in whole ovaries or follicular/luteal tissues from various mammalian species, including humans [55], mice [56]–[58], pigs [59], cattle [60]–[63] and sheep [64] using cloning-based or next generation sequencing strategies [for review see 65–67]. Some studies also identified and characterized miRNAs that are expressed in specific ovarian compartments, including follicular mouse [36], [68] and horse [69] granulosa cells, cow cumulus-oocyte complexes [70], equine follicular fluid [69] and bovine corpora lutea [71]. In addition, other studies reported differences in miRNA expression between different ovarian or follicular compartments. For example, miRNA-503, miRNA-224 and miRNA-383 are expressed almost exclusively in mouse granulosa cells and oocytes [68], [72], whereas a large number of miRNAs are differentially expressed in bovine ovarian cortex, cumulus cells and corpus luteum [60]. Furthermore, a correlation was recently reported between miRNA levels of horse follicular fluid and granulosa cells [69]. Despite these various findings, very little information is currently available about the hormonal regulation of miRNAs in the ovary. One study reported a robust induction of miRNA-21, miRNA-132 and miRNA-212 following *in vivo* stimulation of mouse ovaries with LH/hCG [36]. Moreover, cultured mouse granulosa cells exhibited a robust induction of miRNA-132 and miRNA-212 when challenged with 8BrcAMP [36]. Another *in vitro* study reported up-regulation of 17 miRNAs and down-regulation of 14 miRNAs following 12 h exposure of mouse granulosa cells to FSH [73]. Our studies, while confirming some of these findings, have identified several additional miRNAs whose expression is up- or down-regulated in response to second messenger (cAMP) treatment of rat granulosa cells.

We also examined the cAMP regulation of miRNA expression in MLTC-1 cells, a model cell line of Leydig cells. Treatment of MLTC-1 cells with Bt_2_cAMP for 6 h increased the expression of miRNA-212, miRNA-183, miRNA-132, miRNA-182 and miRNA-96, and inhibited the expression of miRNA-138 and miRNA-19a. To our knowledge this is the first report showing hormone-induced changes in the levels of the above mentioned miRNAs in Leydig cells. Follow-up studies are in progress to more critically examine their potential role in the regulation of testosterone production by Leydig cells. Furthermore, as summarized in Table 2, several genes involved in lipid metabolism and steroidogenesis, whose expression levels are altered by hormones in the adrenal, ovarian granulosa cells and testicular Leydig cell line (MLTC-1), are predicted to be target genes of miRNAs. Ongoing studies are also evaluating the actions of selected hormone responsive miRNAs on potential target genes and secondarily on steroidogenesis. In this context, we have evaluated the effects of miRNA-138, miRNA-132 and miRNA-132/miRNA-214 on the expression of StAR, SREBP-1c and LDLR, respectively, by carrying out 3′UTR luciferase assays. Overexpression of pre-miRNA-132 and pre-miRNA-214 significantly decreased the luciferase activity of the 3′UTR of the SREBP-1c and LDLR reporter containing micRNA-132 and miRNA-214 binding sites, respectively. In contrast, no inhibitory effect of pre-miRNA-138 on the StAR 3′ UTR (with 2 putative binding sites) reporter construct and pre-miRNA-182 on the LDLR 3′UTR (with a single putative binding site) reporter construct was detected.

In conclusion, the current study provides the first comprehensive analysis of hormonal regulation of miRNAs in steroidogenic cells of the adrenal, ovary and testis. The results defined the miRNA expression profiles in rat adrenals in response to treatment with three different hormones (ACTH, 17α-E2 and DEX), and identified several miRNAs that are subject to hormonal regulation in ovarian granulosa cells and testicular Leydig cells. Understanding their actions on potential target genes involved in lipid metabolism should aid greatly in defining the post-transcriptional/post-translational mechanisms by which specific miRNAs may contribute to the regulation of steroidogenesis.
